# Assessment of corrective actions implemented by healthcare facilities following a multicentre observational study on peripheral intravenous catheters

**DOI:** 10.1186/1753-6561-5-S6-P54

**Published:** 2011-06-29

**Authors:** D Verjat-Trannoy, J Tanguy, D Thillard, P Astagneau

**Affiliations:** 1 CCLIN Paris-Nord, Paris, France; 2 ARLIN Haute-Normandie, Rouen, France

## Introduction / objectives

A multicentre practice assessment study on peripheral intravenous catheters (PVC) utilization was carried out in 920 healthcare facilities (HCF) in France in 2010. Breaches in practices were observed particularly for insertion and maintenance of PVC.

The objective was to evaluate the impact of the study practices on implementation of corrective measures.

## Methods

All HCF in Northern France participating in the practices study were requested by electronic way to answer an on-line questionnaire with the following items: protocol of care (creation, reactualization, diffusion, accessibility), organization and practices (formations, working groups, traceability documents, data transmission, monitoring), change of material or products (catheters, gloves, collectors, handrub products, disinfectants, bandages, compresses).

## Results

Overall, 125 HCF of 280 participating in the practices study responded to the questionnaire (45%). They were mainly public hospitals and private clinics (89%). Actualization of care protocols (81%), traceability (59%), and training programs on the insertion and maintenance of the PVC (54%) were the most frequent reported corrective actions. One third of HCF modified disinfectant product or PVC type according to standard recommendations.

## Conclusion

This study showed that direct observation of clinical practices could have a significant impact on safety and quality of PVC use and may reduce infectious risk.

## Disclosure of interest

None declared.

